# Immunotherapy-associated autoimmune hemolytic anemia

**DOI:** 10.1186/s40425-017-0214-9

**Published:** 2017-02-21

**Authors:** Uqba Khan, Farman Ali, Muhammad Siddique Khurram, Awais Zaka, Tarik Hadid

**Affiliations:** 1 Graduate Medical Education, St. John Hospital and Medical Center, Detroit, MI USA; 2 Department of Internal Medicine, Henry Ford Macomb Hospital, Clinton Township, MI USA; 3 Van Elslander Cancer Center, 19229 Mack Ave, Suite 23, Grosse Pointe Woods, MI 48236 USA

**Keywords:** Immunotherapy, Nivolumab, Ipilimumab, Autoimmune hemolytic anemia

## Abstract

**Background:**

Immunotherapy has been widely used in the treatment of several solid and hematologic malignancies. Checkpoint inhibitors have been the forefront of cancer immunotherapy in recent years. Cytotoxic T-lymphocyte-associated protein 4 (CTLA-4) and programmed cell death 1 (PD-1) pathway are the prototypic checkpoint targets for immunotherapy. When combined, CTLA-4 and PD-1 checkpoint inhibitors work synergistically, but with increased probability of toxicity. The following case represents an unusual adverse effect of combined treatment with ipilimumab and nivolumab used for treatment of metastatic melanoma.

**Case presentation:**

A 43-year-old woman with metastatic melanoma presented with severe generalized weakness and fatigue. She has received two cycles of ipilimumab and nivolumab, last administered 3 weeks prior to her presentation. Initial investigations revealed severe anemia with appropriate reticulocytosis, severely elevated lactate dehydrogenase, undetectable haptoglobin level and positive direct coombs test. Patient was diagnosed with severe autoimmune hemolytic anemia secondary to ipilimumab and nivolumab. She was successfully treated with high dose steroids and rituximab.

**Conclusions:**

In our case, we present a rare but serious adverse effect of immunotherapy. We illustrate the clinical presentation and management of immunotherapy associated autoimmune hemolytic anemia. Immunotherapy has revolutionized the treatment of many malignant conditions; therefore, it is imperative for health care professionals caring for cancer patient to be familiar with the adverse effects of immunotherapy, which allow for early recognition and management of these potentially lethal side effects.

## Background

Immunotherapy has been widely used in the treatment of several solid and hematologic malignancies [1]. It has led to paradigm shift in management of advanced cancers with potential for long-term and durable responses [2]. There are several immunotherapy agents approved for use in oncology practice and many others under investigation including chimeric antigen receptor T-cells (CAR T-cells), use of checkpoint inhibitors, interleukin therapy, oncolytic viruses and vaccines. Checkpoint inhibitors have been the forefront of cancer immunotherapy in recent years. Their use have revolutionized the treatment of various malignancies including melanoma, head and neck cancers, lung cancer, renal cell carcinoma, bladder cancer and Hodgkin’s lymphoma. Endogenous immune checkpoints terminate immune response after antigen activation [3]. Cytotoxic T-lymphocyte-associated protein 4 (CTLA-4) and programmed cell death 1 (PD-1) pathway are the two prototypic checkpoint targets for immunotherapy. CTLA-4 and PD-1 checkpoint inhibitors also work synergistically [4] which enhances their efficacy, but with increase risk of adverse effects as well. The following case represents an unusual adverse effect of combined treatment with ipilimumab and nivolumab used for treatment of metastatic melanoma.

## Case presentation

A 43-year-old woman with history of metastatic melanoma presented to emergency room with severe fatigue. Patient stated that she started having generalized weakness and shortness of breath 2 weeks ago. She denied any fever, cough, chest pain or any bleeding episodes. Her history was significant for recently diagnosed metastatic melanoma to brain, liver and right iliac lymph node. B-raf and c-kit mutations were negative. After completing a course of whole brain radiation therapy, she was started on immunotherapy using ipilimumab and nivolumab. She received two cycles of treatment with the last treatment given 3 weeks prior to presentation. Her medical history was significant for hypothyroidism and 20 pack-year smoking. She had significant family history of melanoma in multiple relatives as well. Her medications include hydrocodone, acetaminophen, dexamethasone 4 mg three times a day, levetiracetam and levothyroxine; none of them was recently introduced. She denied using any over-the-counter medications or supplements. Physical examination was unremarkable except for obesity.

Initial laboratory investigations (Table 1) showed white cell count of 8200/mm^3^, hemoglobin of 5.6 gm/dL, platelet count of 122,000/mm^3^ and reticulocyte count of 6.5% (absolute reticulocyte count of 0.11 million/mm^3^). Further work up revealed lactate dehydrogenase of 1406 IU/L (reference normal value: 240 IU/L), total bilirubin of 1.5 mg/dL, haptoglobin of < 10 mg/dL, creatinine of 0.7 mg/dL and positive direct coombs test (Positive for both anti-Ig G and anti-complement 3d). Peripheral smear revealed several spherocytes and multiple polychromatic cells. No schistocytes were present on peripheral smear.

**Table 1:** Laboratory findings at the time of diagnosis of AIHA

Hemoglobin	5.6 gm/dL
Reticulocyte count	6.5%
LDH	1406 IU/L
Haptoglobin	<10 mg/dL
Total Bilirubin	1.5 mg/dL
Coombs Test	Positive for both anti-Ig G and anti-complement 3d
Peripheral smear findings	Spherocytes and polychromatic cells

**Fig. 1:**
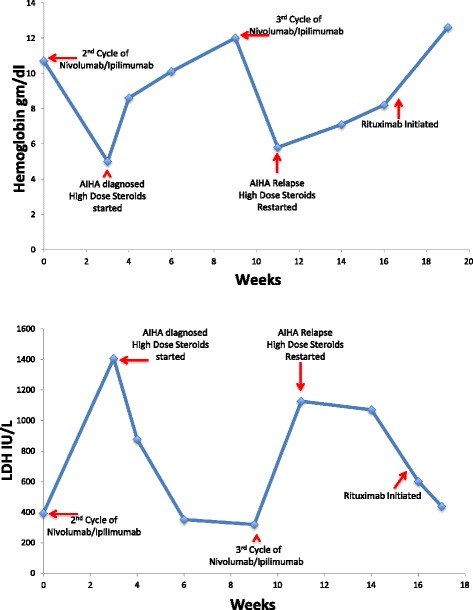
Graph for hemoglobin and LDH versus time

Patient was diagnosed with autoimmune hemolytic anemia (AIHA) secondary to immunotherapy with ipilimumab and nivolumab. She was given multiple blood transfusions and started on pulse dose steroids using 1000 mg of intravenous methylprednisolone daily for 3 days. She was then transitioned to oral prednisone 1 mg/kg daily and was tapered over a period of several weeks. Her hematological parameters improved gradually over a period of 2 months. Immunotherapy was held during this period. Once her hemoglobin normalized, she was re-challenged with ipilimumab and nivolumab. She developed hemolytic anemia again after administration of immunotherapy. She was re-treated with similar steroid regimen. Due to slow response to steroids and concern for steroid related side effects, rituximab was added at a weekly dose of 375 mg/m^2^ for 4 weeks. She responded well to this treatment and at the time of last follow up her hemoglobin normalized. Response to treatment is demonstrated in Fig. 1. Immunotherapy was put on hold again. During this period, imaging studies showed improvement in brain lesions and stable disease elsewhere.

## Discussion

Melanoma is a highly malignant tumor of skin. It is the fifth most common tumor in men and seventh in women in United States [5]. The prognosis of metastatic melanoma is poor with a 5-year survival of only 15–20%. There is great progress made in recent years in the management of advanced melanoma resulting in approval of several drugs by Food and Drug Administration (FDA). Ipilimumab and nivolumab are two of the several drugs recently approved for metastatic melanoma (http://www.fda.gov/Drugs/InformationOnDrugs/ApprovedDrugs/ucm465274.htm). Ipilimumab is a CTLA-4 inhibitor and nivolumab is a PD-1 inhibitor, both results in enhanced anti-tumor immune response [6].

Approximately 50% of AIHA cases are idiopathic [7] but in the remaining cases, underlying inciting factor can be found. Common etiology includes underlying malignancy, autoimmune disorders, drugs and infections [8]. Diagnosis of AIHA is relatively easy given the constellation of laboratory findings of normocytic or macrocytic anemia, reticulocytosis, low serum haptoglobin levels, elevated LDH level, increased indirect bilirubin level and a positive direct antiglobulin test. Once the diagnosis is established, it is important to rule out any secondary causes as managing the underlying cause can indirectly improve the AIHA, particularly in drug-induced hemolytic anemia [7]. Initial treatment of AIHA involves high dose steroids with slow taper. Steroids work primarily by inducing immunosuppression resulting in reduction of autoantibodies production. Rituximab is usually reserved for refractory and relapsed cases. Rituximab is a chimeric monoclonal antibody that causes apoptosis of B-lymphocytes by binding to CD20 positive B-lymphocytes. Depletion of B-lymphocytes lead to reduction in autoantibodies, inflammatory cytokines and T-cell activation.

Due to increasing use of immunotherapy, there is growing number of reports of AIHA related to checkpoint inhibitors, primarily anti-PD1 as depicted in Table 2 [9–13]. Interestingly, no AIHA was reported in patients who received the combination of ipilimumab and nivolumab in the medical literature. Our case was different from reported cases in number of ways: first, it documents the recurrence of AIHA after re-challenge of combined immunotherapy drugs; second, unlike the other cases, we propose rituximab as a treatment choice for relapsed and refractory cases of immunotherapy-induced AIHA; third, none of the other cases reported AIHA due to combined immunotherapy. It can be learned from our experience that rituximab can also be successfully used for relapsed or resistant cases of immunotherapy induced AIHA. It is unknown which of the two drugs caused AIHA in our patient, however both drugs have been reported to be implicated in causing AIHA as previously described in Table 2. The underlying mechanism of immunotherapy-induced hemolytic anemia is unconfirmed but generally drug induced AIHA is categorized into drug-independent (via auto-antibodies) and drug-dependent antibodies causing immune hemolytic anemia. A drug-dependent antibody activates a response only while the drug is present. In contrast drug independent antibodies, are capable of creating an autoimmune response in the absence of the offending drug [14]. It is not clearly understood why autoantibodies production begins but a number of mechanisms have been proposed including defective control of IgG auto-reactivity by autologous IgM and alteration of T-cell function [15–18].

**Table 2:** Reported cases of immunotherapy associated AIHA

Case Report	Type of immunotherapy used	No of prior cycles	Type of malignancy	Mediator IgG or C3	Treatment of AIHA	Immunotherapy Re-challenge	Other possible association for AIHA
Schwab KS. et al. [9]	Nivolumab	8	Metastatic SCC of skin	IgG, C3	Steroids	No	CLL
Tardy MP. et al. [10]	Nivolumab	2	Hodgkin’s lymphoma	IgG	Steroids	Yes, 6 more injections. No recurrence of AIHA	None
Kong BY. et al. [11]	Initially Ipilimumab then patient was started on Nivolumab	4	Metastatic melanoma	IgG	Steroids	No	Patient had positive direct anti-globulin test before starting Nivolumab.
Palla AR. et al. [12]	Nivolumab	2	Metastatic lung cancer	C3	Steroids	No	None
Simeone E. et al. [13]	Ipilimumab	3	Metastatic melanoma	Unknown	Steroids	Unknown	None
Simeone E. et al. [13]	Ipilimumab	3	Stage III melanoma	Unknown	Steroids	Unknown	None
Simeone E. et al. [13]	Ipilimumab	4	Metastatic melanoma	Unknown	Steroids	Unknown	None

## Conclusions

AIHA can be life threatening if not treated promptly. It is always prudent to exclude any inciting factor, particularly drugs because cessation of the drug is often sufficient to resolve the hemolysis. Immunotherapy agents have been approved in several solid and hematologic malignancies [19], therefore, it is important for all health care professionals caring for cancer patients to recognize the adverse effects of immunotherapy, especially AIHA as depicted in our case.

## Abbreviations

AIHA: Autoimmune hemolytic anemia
CAR T-cells: Chimeric antigen receptor T-cells
CLL: Chronic lymphocytic leukemia
CTLA-4: Cytotoxic T-lymphocyte-associated protein 4
FDA: Food and Drug Administration
PD-1: Programmed cell death 1
PD-L1: Programmed death-ligands 1
SCC: Squamous cell carcinoma
